# Chemical Composition of Five Lamiaceae Essential Oils and Their Insecticidal and Phytotoxic Activity

**DOI:** 10.3390/plants13162204

**Published:** 2024-08-09

**Authors:** Tianhao Pei, Yijin Zhao, Xudong Huang, Yinyue Zhao, Liudan Pan, Lingwei Wang, Hexin Gao, Meng-Lei Xu, Yu Gao

**Affiliations:** 1 College of Plant Protection, Jilin Agricultural University, Changchun 130118, China; 2 Key Laboratory of Soybean Disease and Pest Control, Ministry of Agriculture and Rural Affairs, Changchun 130118, China; 3 Dalian City Investment Asset Management Co., Ltd., Dalian 116021, China; 4 Institute of Food Crops, Yunnan Academy of Agricultural Sciences, Kunming 650205, China; 5 College of Food Science and Engineering/State Key Laboratory of Supramolecular Structure and Materials, Jilin University, Changchun 130062, China

**Keywords:** Lamiaceae, essential oil, insecticidal activity, *Thrips flavus*, herbicidal effect

## Abstract

The Lamiaceae family is widely distributed worldwide. In this study, we investigated the insecticidal activity of five Lamiaceae essential oils against *Thrips flavus* Schrank and the phytotoxic activity against *Glycine max* (L.) Merr., *Zea mays* L., *Portulaca oleracea* L., and *Echinochloa oryzoides* (Ard.) Fritsch. Then, the chemical composition of the five essential oils was analyzed by using gas chromatography–mass spectrometry (GC-MS). The five Lamiaceae essential oils were melissa, basil, rosemary, negundo chastetree, and salvia. The main constituents of the five Lamiaceae essential oils were preliminarily determined to be as follows: α-pinene and 1,8-cineole in the rosemary essential oil; *β*-pinene, *γ*-terpinene, and *d*-limonene in the negundo chastetree essential oil; *β*-cadinene and isolongifolen-5-one in the melissa essential oil; 5-allylguaiacol in the basil essential oil; and isopropyl myristate, linalyl acetate, and linalool in the salvia essential oil. Using a bioassay, it was found that, among the five essential oils, the melissa essential oil exhibited the lowest LC_50_ value, which was 0.18 mg/mL, and the salvia essential oil exhibited the highest LC_50_ value, which was 0.42 mg/mL. The control efficacy of the five essential oils significantly increased with time and concentration in pot experiments. The negundo chastetree, basil, rosemary, and salvia essential oils at 900.00 g a.i.·hm^−2^ showed high control efficacy against *T. flavus*, with values higher than 90%. Female thrips were attracted to the negundo chastetree essential oil. The five essential oils were also tested for their effects on the germination rate, germination potential, germination index, and shoot length of *G. max*, *Z. mays*, *P. oleracea*, and *E. oryzoides*. The basil essential oil significantly inhibited the germination of *P. oleracea*, with germination at a concentration of 1.0 mg/mL being only 11.11 ± 5.09%. This study provides a reference for the development of botanical pesticides to control *T. flavus*, crops, and weeds.

## 1. Introduction

Lamiaceae plants belong to the order Labiata, which includes approximately 245 plant genera and 7886 species and is widely distributed worldwide, with most of the order originating from Asia, Africa, and Europe [1]. Essential oils (EOs) derived from Lamiaceae possess a range of medicinal benefits, including antioxidant and anti-inflammatory properties, as well as wound-healing and anti-cancer potential, making them valuable assets in the field of medicine [2]. Additionally, these EOs exhibit potential as botanical herbicides and alternatives to artificial preservatives in agriculture and food science [3,4]. The major components of most Lamiaceae EOs are terpenoids, such as 1,8-cineole, linalool, terpinene, thymol, *α*-pinene, and *β*-pinene, which have been identified in most species [5]. The insecticidal activity of Lamiaceae EOs against agricultural, sanitary, and storage pests has been previously demonstrated. Their insecticidal activities include fumigation, repellency, and larvicidal activity [6,7,8,9]. A study reported that the mortality rate of *Sitophilus granarius* L. (Coleoptera: Curculionidae) reached 99.59% after 24 h of treatment with *Ocimum basilicum* L. essential oil (EO) [6]. *Origanum majorana* L. and *Satureja thymbra* L. EOs exhibited repellent activity against *Aedes albopictus* (Diptera: Culicidae) [7]. *Thymus zygis* L. exhibited significant ovicidal and larvicidal activity against *Plutella xylostella* (L.) (Lepidoptera: Plutellidae) [8]. The repellency rate and mortality rate of *Mentha longifolia* L. EO on *Aphis craccivora* Koch (Hemiptera: Aphididae) were reported to be 84.37% and 80.66%, respectively, at a lethal concentration of 8 µL/mL. The LC_50_ value under the action of contact was 1.848 µL/mL [9]. *Lavandula dentata* L. EO was reported to be used as an alternative in the control of *Anticarsia gemmatalis* (Hübner, 1818) in soybean pest management, and the lethality rate could reach up to 100% [10]. Therefore, EOs derived from Lamiaceae species show promising potential as biopesticides. The potential of Lamiaceae EOs should be fully explored and exploited for agricultural pest control, particularly to control pest thrips.

*Thrips flavus* Schrank (Thysanoptera: Thripidae) is a phytophagous pest widely distributed on the Eurasian continent [11,12,13]. It is a quarantine pest in Slovakia [13,14]. Soybean is a suitable host plant for *T. flavus*. Soybean leaves attacked by *T. flavus* develop spots, leaf curling, chlorosis, and wilting [11,15]. The time required for *T. flavus* to complete one generation on soybeans is approximately 20 days [16], which poses a serious potential threat to soybean production in China [17]. Temperature is an important environmental factor that affects population dynamics, and it is a key consideration in integrated thrips management [18,19]. There is a linear relationship between temperature and the rate of *T. flavus* development from eggs to adults, and female thrips have the highest fecundity at 19 °C [11]. This pest has been reported to transmit the tomato spotted wilt virus (TSWV) [20]. However, there are no reports on whether *T. flavus* is a vector for the transmission of soybean virus diseases; nevertheless, the risk should not be ignored. Thiamethoxam and imidacloprid are currently the most promising chemical insecticides against *T. flavus* [21]; however, the long-term irrational use of chemical pesticides will result in the development of resistance to common insecticides in thrips, and the level of resistance is constantly increasing [22]. The extensive use of chemical pesticides also affects organic farming, causing problems with pesticide residues in food [23]. The development of alternative chemical pesticides to address the resistance to conventional chemical insecticides is currently an important issue in thrip control.

We aimed to determine the chemical composition of five EOs from the Lamiaceae family using gas chromatography–mass spectrometry (GC-MS). The insecticidal activity of and the *T. flavus* olfactory response to the EOs were measured using the leaf-dipping method and the olfactory behavior test, respectively. This study could serve as a basis for the control of thrips pests and the development of botanical pesticides against *T. flavus* and weeds.

## 2. Results

### 2.1. Chemical Composition Analysis

Twenty-one compounds were detected in the salvia essential oil. The most abundant compounds included isopropyl myristate (28.4%), linalyl acetate (19.3%), linalool (14.5%), and benzyl benzoate (12.3%) (Table 1).

A total of 19 compounds were detected in the rosemary essential oil, with *α*-pinene, 1,8-cineole, and camphene being the most abundant, accounting for 26.2%, 25.9%, and 10.4%, respectively (Table 2).

Twelve compounds were detected in the basil essential oil. The most abundant compounds were 5-allylguaiacol, accounting for 50.2%, and Bicyclo [5.2.0] nonane, 4-ethenyl-4,8,8-trimethyl-2-methylene- and *α*-copaene, accounting for 21.9% and 12.1%, respectively (Table 3).

Eleven compounds were detected in the negundo chastetree essential oil. The most abundant compound was *β*-pinene, constituting 32.9% of the total compounds, followed by *d*-limonene (27.8%) and *γ*-terpinene (16.9%) (Table 4).

Thirteen compounds were detected in the melissa essential oil. Isolongifolen-5-one was the most abundant compound, accounting for 29.6% of all the compounds, followed by *β*-cadinene (25.5%) and aristolone (12.1%) (Table 5).

### 2.2. Laboratory Bioassay

The salvia essential oil had the highest LC_50_ (lethal concentration 50) among the five EOs, which was 0.42 mg/mL (Table 6). The melissa essential oil had the lowest LC_50_ value at 0.18 mg/mL, followed by the negundo chastetree, basil, and rosemary essential oils, with LC_50_ values of 0.34 mg/mL, 0.35 mg/mL, and 0.37 mg/mL, respectively. A comparison of the 95% confidence intervals revealed an overlap between those of the melissa and basil essential oils, but no overlap with those of the other EOs. This suggests that the insecticidal toxicity of the melissa essential oil was significantly higher than that of the other EOs.

### 2.3. Pot Experiments

**Figure 1:**
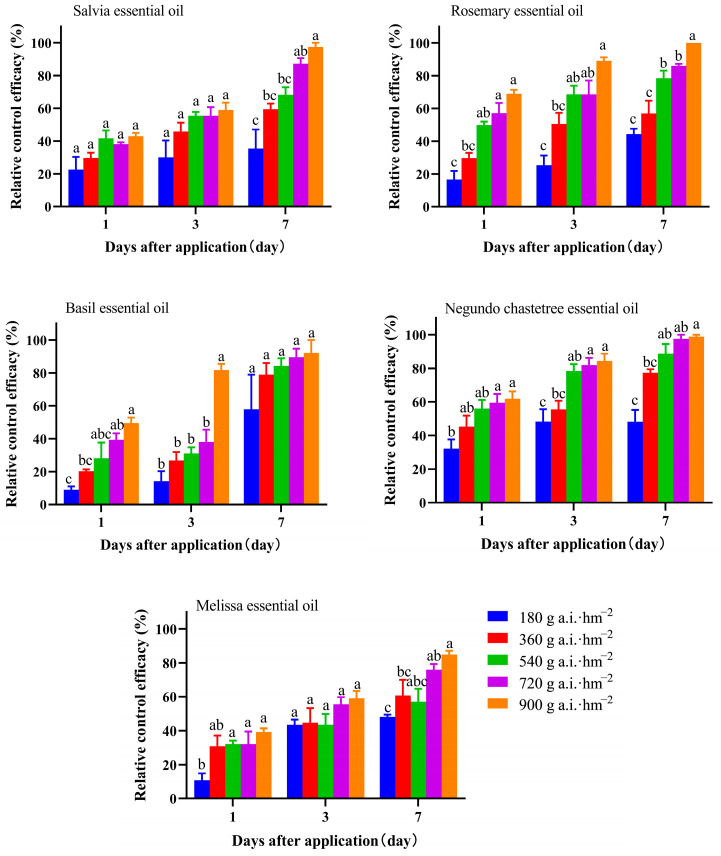
Relative control efficacy of the five EOs in a pot experiment. For the same essential oil, different lowercase letters following the data in the same column indicate significant differences in the relative control efficacy of the five doses (*p* < 0.05).

The pot experiments revealed a significant increase in control efficacy with increasing concentration (Figure 1). After one day of application, among the tested EOs, there were significant variations in the control efficacy of the melissa (*p* = 0.0129), negundo chastetree (*p* = 0.0167), basil (*p* = 0.0020), and rosemary essential oils (*p* < 0.0001). However, the salvia essential oil did not show significantly different control efficacies (*p* = 0.0615). After three days of application, there was a significant variation in the control efficacy of the negundo chastetree (*p* = 0.002), basil (*p* = 0.0002), and rosemary essential oils (*p* = 0.0003). However, there were no significant differences in the control efficacy of the melissa essential oil (*p* = 0.2283) or salvia essential oil (*p* = 0.0553). After seven days of application, there were significant differences in the control efficacy of the melissa (*p* = 0.0063), negundo chastetree (*p* = 0.0007), rosemary (*p* < 0.0001), and salvia essential oils (*p* = 0.0002). No significant difference was observed in the control efficacy of the basil essential oil (*p* = 0.4737).

There were significant differences in the control efficacy of the five EOs at the same treatment duration and dose. The control efficacy of the five EOs exhibited significant differences at 180.00 g a.i.·hm^−2^ after three days of application (*p* = 0.0495). However, no significant difference was observed after one day (*p* = 0.0822) or seven days (*p* = 0.6557) of application. The basil essential oil treatments had the highest control efficacy of 57.89% ± 21.05% at 180.00 g a.i.·hm^−2^, whereas the efficacy of the other EOs was <50%. A significant difference was also observed at 360.00 g a.i.·hm^−2^ after one day of application (*p* = 0.0354). The control efficacy of the negundo chastetree essential oil (45.24% ± 6.63%) was significantly higher than that of basil (20.22% ± 1.12%). No significant differences were observed three days (*p* = 0.0730) or seven days (*p* = 0.0905) after application. The control efficacy exhibited significant differences at 540.00 g a.i.·hm^−2^ after one day (*p* = 0.0371), three days (*p* = 0.0002), and seven days (*p* = 0.0378) of application. The control efficacy of the negundo chastetree essential oil treatments was significantly higher than that of the other EOs after one day (55.95% ± 5.19%), three days (78.31% ± 4.17%), and seven days (88.61% ± 5.80%) of application. The control efficacy of the five EOs showed significant differences at 720.00 g a.i.·hm^−2^ after one day (*p* = 0.0142), three days (*p* = 0.0057), and seven days (*p* = 0.0403) of application. There were significant differences in the control efficacy of the five EOs at 900.00 g a.i.·hm^−2^ after one day (*p* = 0.0001) and three days (*p* = 0.0007), but no significant difference was observed after seven days (*p* = 0.0860). The lowest control efficacy of the five EOs at 900.00 g a.i.·hm^−2^ after seven days was observed in the case of the melissa essential oil treatment (84.81% ± 2.19%); the control efficacies of the other treatments were >90%.

The control efficacy of the five EOs increased significantly with time and dose. The negundo chastetree, basil, rosemary, and salvia essential oils at a concentration of 900.00 g a.i.·hm^−2^ exhibited high control efficacy against *T. flavus*, with rates of 98.73% ± 1.27%, 92.11% ± 7.89%, 100%, and 97.47% ± 2.53%, respectively. The control efficacy of the highest dose of the basil essential oil was not significantly different from that of the other doses.

### 2.4. Olfactory Behavior Test

**Figure 2:**
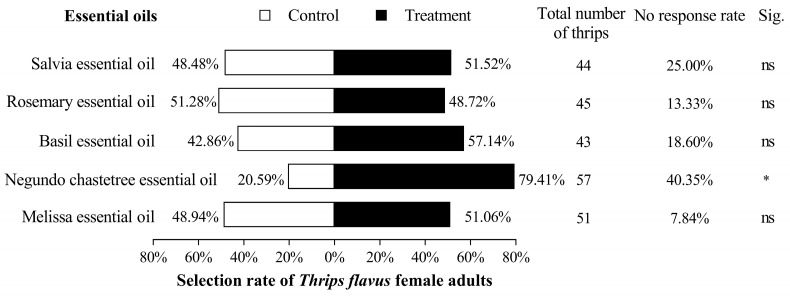
Olfactory behavioral response of female adult *Thrips flavus* to the five EOs. “ns” indicates no significant difference between them. “*” indicates a significant difference (*p* < 0.05) between the control and the treatment. The same below.

The seduction rates of the melissa, negundo chastetree, basil, rosemary, and salvia essential oils in adult females were 51.06% (*χ*^2^ = 0.014, *p* = 0.904), 79.41% (*χ*^2^ = 4.948, *p* = 0.026), 57.14% (*χ*^2^ = 0.530, *p* = 0.467), 48.72% (*χ*^2^ = 0.018, *p* = 0.892), and 51.52% (*χ*^2^ = 0.023, *p* = 0.880), respectively. *T. flavus* female adults were only attracted to the negundo chastetree essential oil and not the other four EOs (Figure 2).

**Figure 3:**
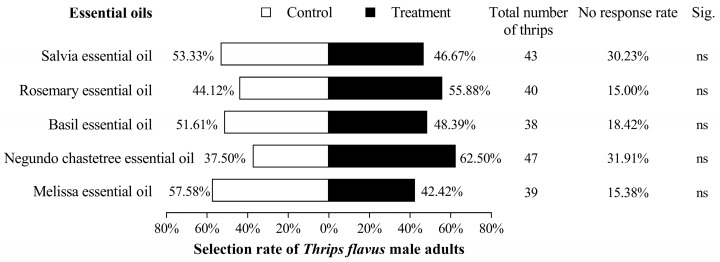
Olfactory behavioral response of male adult *Thrips flavus* to five EOs.

The seduction rates of the melissa, negundo chastetree, basil, rosemary, and salvia essential oils against male adults were 42.42% (*χ*^2^ = 0.570, *p* = 0.450), 62.50% (*χ*^2^ = 1.521, *p* = 0.218), 48.39% (*χ*^2^ = 0.025, *p* = 0.875), 55.88% (*χ*^2^ = 0.351, *p* = 0.553), and 46.67% (*χ*^2^ = 0.103, *p* = 0.749), respectively. None of the five EOs had a significant attractive or repellent effect on *T. flavus* (Figure 3).

### 2.5. Phytotoxic Activity

The salvia essential oil had a significant effect on the germination rate, germination potential, and shoot length of *G. max* (Table 7). For *G. max*, the germination rates of 0.2 mg/mL, 0.4 mg/mL, and 0.8 mg/mL were significantly lower than those of the control (*F* = 13.749, *p* < 0.001), and the germination potential also showed the same phenomenon (*F* = 12.570, *p* < 0.001), but both reached more than 95%. The shoot lengths of 0.2 mg/mL, 0.4 mg/mL, 0.6 mg/mL, and 1.0 mg/mL were significantly lower than those of the control (*F* = 8.569, *p* < 0.001). The shoot length of *P. oleracea* after the treatment with 0.8 mg/mL and 1.0 mg/mL of the salvia essential oil was significantly lower than that after the treatment with 0.2 mg/mL (*F* = 6.336, *p* = 0.004).

The negundo chastetree essential oil had a significant effect on the germination potential of *G. max* and *Z. mays* and on the shoot length of *P. oleracea*. However, different concentrations of the negundo chastetree essential oil had no significant effect on the germination rate or germination index of the four plants (Table 8). The germination potential of *G. max* at 1.0 mg/mL was significantly lower than that at 0.2 mg/mL, 0.4 mg/mL, and 0.6 mg/mL and the control (*F* = 6.109, *p* = 0.005). The germination potential of *Z. mays* at 1.0 mg/mL was significantly lower than that at 0.2 mg/mL (*F* = 4.164, *p* = 0.020). The shoot length of *P. oleracea* at 0.6 mg/mL and 0.8 mg/mL was significantly lower than that at 0.2 mg/mL and 1.0 mg/mL (*F* = 7.255, *p* = 0.002).

The basil essential oil had a significant effect on the germination potential and shoot length of *G. max*; the germination index of *Z. mays*; all four indices of *P. oleracea*; and the germination rate, germination potential, and germination index of *E. oryzoides* (Table 9). The germination potential of *G. max* at 0.2 mg/mL was significantly lower than that of the others (*F* = 3.874, *p* = 0.025). The shoot lengths of *G. max* at 0.8 mg/mL and 1.0 mg/mL were significantly shorter than those of the control (*F* = 4.032, *p* = 0.022). Significant effects on the germination index of *Z. mays* were observed after the treatment with 0.8 mg/mL and 1.0 mg/mL, and it was significantly lower than that of the control (*F* = 5.108, *p* = 0.010). The germination potential at 0.6 mg/mL, 0.8 mg/mL, and 1.0 mg/mL was significantly lower than that of the control; the lowest was observed at 1.0 mg/mL, being only 11.11 ± 5.09% (*F* = 70.002, *p* < 0.001). The germination potential at 0.6 mg/mL, 0.8 mg/mL, and 1.0 mg/mL was significantly lower than that of the control (*F* = 52.046, *p* < 0.001), and the germination index was also significantly lower than that of the control (*F* = 186.614, *p* < 0.001). The shoot length after the treatment at 0.2 mg/mL was significantly longer than that after the treatment with the other concentrations and of the control (*F* = 509.312, *p* < 0.001). The germination rate (*F* = 3.606, *p* = 0.032), germination potential (*F* = 5.193, *p* = 0.009), and germination index of *E. oryzoides* (*F* = 9.552, *p* < 0.001) at 1.0 mg/mL were significantly lower than those at 0.2 mg/mL and of the control.

The rosemary essential oil had no significant effect on the germination rate or germination potential of the four plants, but there were some differences in the shoot lengths of *P. oleracea* and *G. max* (Table 10). The shoot length of *G. max* was significantly reduced by the treatments at 0.6 mg/mL and 1.0 mg/mL (*F* = 7.167, *p* = 0.003). The shoot length of *P. oleracea* was significantly lower at 1.0 mg/mL than at 0.4 mg/mL and 0.6 mg/mL, but not significantly different from that of the control (*F* = 25.475, *p* < 0.001). The germination index of *E. oryzoides* at 1.0 mg/mL was significantly lower than that at the other concentrations and of control (*F* = 3.321, *p* < 0.041).

The melissa essential oil affected the germination index of *G. max*; the germination rate, germination potential, and shoot length of *Z. mays*; and the shoot length of *P. oleracea* (Table 11). The germination index of *G. max* at 0.2 mg/mL was significantly higher than that at 0.6 mg/mL (*F* = 4.213, *p* = 0.019). The germination rate of *G. max* at 0.2 mg/mL was significantly higher than that at 0.6 mg/mL (*F* = 5.661, *p* = 0.007). The germination index of *G. max* at 0.2 mg/mL was significantly higher than that at 0.6 mg/mL and 1.0 mg/mL (*F* = 4.628, *p* = 0.014). The shoot length of *Z. mays* at 0.4 mg/mL and the control was significantly longer than that at 0.2 mg/mL (*F* = 6.641, *p* = 0.003). The shoot length of *P. oleracea* became significantly shorter with increasing concentrations (*F* = 126.364, *p* < 0.001).

## 3. Discussion

Essential oils have been shown to be effective in agricultural pest management, particularly in the control of thrips. In this study, the insecticidal toxicity of five Lamiaceae EOs against thrips was tested using the leaf-dipping method. The melissa (LC_50_ = 0.18 mg/mL), negundo chastetree (LC_50_ = 0.34 mg/mL), and basil essential oils (LC_50_ = 0.35 mg/mL) exhibited high insecticidal toxicity against *T. flavus*. Previous studies have shown that several of the EOs involved in this study have diverse biological activities against pests. Melissa essential oil was reported to have antifeedant activity and strong fumigant toxicity against *Tribolium castaneum* Herbst (Coleoptera: Tenebrionidae) (LC_50_ of 19.418 μL/L air after 24 h of treatment), and also showed toxicity against the larvae and pupae [24]. The LC_50_ for the larvae of *Aedes vittatus* Bigot and *Anopheles maculates* Theobald (Diptera: Culicidae) was 22.19 μg/mL and 26.09 μg/mL, whereas the LC_50_ for the pupae was 101.15 μg/mL and 94.69 μg/mL, respectively [25]. The adsorption of basil essential oil using modified clay effectively prolonged the toxicity of the EO against the corn weevil, retaining 40% of the insecticidal activity 30 days after application [26]. Rosemary essential oil exhibited fumigant activity against *Thrips palmi* Karny (Thysanoptera: Thripidae), causing 100% lethality against *T. palmi* after 24 h at a concentration of 80.13 mg/L air. The LC_50_ was determined to be 17.68 mg/liter of air, indicating that the essential oil’s fumigant toxicity was significantly greater than that of dichlorvos, methamido-abamectin benzoate, spinosad, and thiamethoxam [27]. Rosemary essential oil was found to be safe for the natural enemies of the western flower thrips *Frankliniella occidentalis* (Pergande) (Thysanoptera: Thripidae). The sub-lethal concentrations of rosemary oil were found to have little effect on the life table parameters of the predacious mite *Amblyseius swirskii* Athias-Henriot (Acari: Phytoseiidae) [28]. The combination of salvia and thymus essential oils showed a significant synergistic effect on *F. occidentalis*, and its lethality against thrips was significantly higher than that of a single essential oil application [29].

EOs can cause changes at different levels in pests. The effects of the EO of *Ocimum gratissimum* L. may be related to its inhibitory effect on acetylcholinesterase and butyrylcholinesterase enzymes [30]. After the treatment with melissa essential oil at the LC_50_ concentration, the reactivity of superoxide dismutase (SOD) and catalase (CAT) in *T. castaneum* increased, whereas the levels of glutathione (GSH) decreased [31]. These findings suggest that the toxic effects of EOs on insects are related to changes in enzyme activity. Molecular docking assays revealed that the *p*-cymene and thymol in essential oils are structurally similar and bind to the AChE active site mainly through hydrophobic interactions and that thymol can bind to the hydrogen bond in the Tyr 374 position [32]. At the cellular level, the EOs of *Thymus vulgaris* (L.) and *Lavandula angustifolia* (Mill.) caused the necrosis of intestinal cells, cellular alterations, etc., in the fifth-instar larvae of *Thaumetopoea pityocampa* Den. & Schiff. (Lepidoptera: Notodontidae) [33].

In general, the dose–response curves for bioassays are right-skewed, i.e., the increase in response rate per unit dose increase at lower doses is higher than the increase in response rate per unit dose increase at higher doses. When the dose or concentration is logarithmically transformed, the transformed dose–response curve has a normalized distribution. As the response ratio (e.g., the mortality of the test insects) travels in a cumulative time curve in relation to the logarithm of the concentration, it is usually necessary to carry out an appropriate statistical transformation to transform this response ratio into a straight line. In this study, we found that the logarithm of the mortality rate of the test organisms versus the concentration was in the form of a cumulative time curve, which is consistent with the above pattern. Thus, the Data Processing System (DPS) was used to transform the relationship into a straight line to facilitate the subsequent calculations.

The chemical components of the five EOs identified in this experiment are consistent with those of a previous study, being representative compounds of Lamiaceae EOs [5], but the contents varied. Esmaeili and Rohani found that cedrane was the main compound in melissa essential oil [34]. Kobenan et al. reported that the most abundant compounds in basil essential oil were *p*-cymene and thymol [30], which is inconsistent with this study. 1,8-cineole, α-pinene, and camphor were found to be the main components of an essential oil from the eastern region of Morocco [35]. Another previous study found that the main components of salvia essential oil included α-terpinyl acetate, *d*-camphor, and linalool [36], which is consistent with our results. These differences in compounds may be caused by the source organ [37], plucking season [38], or extraction method [39], among other factors. A study reported that 5-allylguaiacol, comprising 50.18% of basil essential oil, was the main component of *Apium graveolens* L. (Apiaceae) and *Syzygium aromaticum* (L.) Merr. & L. M. Perry (Myrtaceae) hexane extract [40,41]. This compound showed toxicity against *Aedes aegypti* L. (Diptera: Culicidae) larvae [42]. Isopropyl myristate is mostly found in the behavioral modulation of insects [43,44]. Linalyl acetate has been reported to have contact and fumigant activities against pests [44,45]. Pinenes (enantiomers of *α*- and *β*-) have shown a strong toxic effect against the mosquito larvae of *Culex pipiens* (Diptera: Culicidae), as well as EOs obtained from the fruit peels of orange and lemon, which contain a high proportion of limonene and lower quantities of *p*-menthane molecules depending on the genotype and oil composition [46]. *β*-caryophyllene, terpinolene, and linalool were less detected in our study compared with what is typically found in EOs. The insecticidal activities of these compounds, as the main components of Lamiaceae plants, have been confirmed and identified in previous studies [47,48,49,50]. This indicates that the toxicity of EOs may arise from the combined effects of different components [51,52]. The compounds found at high levels in EOs are not necessarily substances that determine the insecticidal activity of the EOs. The EO of *Amomum villosum* Loureiro (Zingiberaceae), which consists of bornyl acetate, camphor, camphene, and limonene, showed contact toxicity against *T. castaneum* and *Lasioderma serricorne* (Fabricius) [53]. 1-octen-3-ol, p-cymene, and 3-octanol are trace components in the EO of *Elsholtzia densa* Benth., but it showed fumigant toxicity against *L. serricorne* [54].

The Y-tube olfactory behavioral test revealed that *T. flavus* female adults were only attracted to the negundo chastetree essential oil. Moon et al. reported similar results indicating that the fourth-instar nymphs of *Lycorma delicatula* (White) (Hemiptera: Fulgoridae) exhibited a significant attraction toward *Mentha spicata* L. (Lamiaceae) EO at 5 μL up to 90.9% [55]. Negundo chastetree is an important medicinal plant. Zeb et al. reported that compounds such as vitexdoin A are the main chemical constituents of negundo chastetree leaf and branch extracts [56]. However, studies on the insecticidal activity of negundo chastetree essential oil have not been reported. The most abundant components of the negundo chastetree essential oil were found to be *β*-pinene *d*-limonene, and *γ*-terpinene. *β*-pinene is attractive to *Dioryctria abietella* (Denis & Schiffermüller) (Lepidoptera: Pyralidae) [57] and elicits a significant electrophysiological response in the female antennae of *Ceratitis capitata* (Wiedemann) (Diptera: Tephritidae) [58]. *Aphytis melinus* DeBach (Hymenoptera: Aphelinidae) was primed with 20 μL/mL of *d*-limonene, with the former being a natural parasitic enemy of *Aonidiella aurantii* (Maskell) (Hemiptera: Diaspididae) [59]. Appropriate proportions of *γ*-terpinene and limonene can significantly attract more *Leptocybe invasa* Fisher & La Salle (Hymenoptera: Eulophidae) in the field [60]. These results indicate that the main chemical components of EOs have the potential to be used as attractants. Some of these main components are identical to the various pheromones of pests in nature, which can attract pests and influence mating behavior [59,61]. Notably, the rosemary essential oil also contained *β*-pinene (1.63%) and *γ*-terpinene (0.83%), whereas the salvia essential oil contained *β*-pinene (0.12%). However, these two components constituted a relatively small percentage of the two EOs. The attraction of the negundo chastetree essential oil to *T. flavus* may be due to the combined action of the three main chemical components. Other compounds in much smaller quantities may also be involved in this process, which needs to be confirmed in further studies.

In this study, the five EOs were tested for their efficacy against thrips by diluting and spraying them onto soybean plants. However, the direct spray method does not fully exploit the potential effects of EOs in the field or during actual production processes against thrips [62]. EOs have been reported to have fumigant activity against thrips, and they can be fully exploited in greenhouses as an alternative to chemical insecticides [63,64]. Combining EOs with natural enemies can be equally effective in controlling pests. In a previous study, the LC_30_ concentration of rosemary essential oil in combination with *A*. *swirskii* had the potential to serve as an alternative to chemical pesticides against *F. occidentalis* [28]. In this study, the melissa essential oil (LC_50_ = 0.18 mg/mL) was the most toxic in the bioassay, but it was less effective in the pot experiments (84.81% ± 2.19% at 900.00 g a.i.·hm^−2^ after seven days of application), which is most likely due to the rapid volatilization of the oil after being sprayed onto the soybean plants. In a previous study, the nano-embedded encapsulation of EOs into a chitosan matrix enhanced their bioefficacy and demonstrated a favorable toxicological profile for non-target mammals [30]. The incorporation of alginate and methyl cellulose polymers in the EOs of *Thymus serpyllum* L. and *Satureja montana* L. significantly improved the repellency of the EOs against *F. occidentalis* [65]. These studies provide ideas for the development of EO preparations and their applications. The selection of appropriate embedding technology and essential oil carriers can effectively enhance the insecticidal effects and prolong the duration of the action of EOs [26,65,66]. Therefore, future research should continue to investigate the bioactivity of these EOs against *T. flavus*. Simultaneously, there is a need to develop a suitable formulation to maximize the efficacy of EOs and adapt it to the various production requirements to enhance the insecticidal effect of EOs.

*G. max* and *Z. mays* are important economic crops. *P. oleracea* and *E. oryzoides* are common weeds in agricultural fields. They are also important host plants for Thysanoptera pests. Testing the Lamiaceae EOs’ toxicity to the two crops and weeds is conducive to identifying safe EOs. Regarding the Lamiaceae EOs tested in this study, their phytotoxicity and inhibitory effects on weeds have also been reported in some cases. The application of rosemary essential oil at a concentration of 5.6 mL/L resulted in a notable inhibition of the length, dry weight, and survival of both the shoots and roots of *Acacia saligna* (Labill.) H.L.Wendl. (Fabaceae) seedlings [67]. The embedding technique was employed for treatment with rosemary essential oil, resulting in a notable reduction in the germination, root length, and leaf area of *Amaranthus retroflexu*s L. (Amaranthaceae) and *Rhaphanus sativus* L. (Brassicaceae) as the concentration of the EO increased [68]. The germination and subsequent growth of *Cucumis sativus* L. (Cucurbitaceae) and *Solanum lycopersicum* L. (Solanaceae) were not significantly affected by the application of different concentrations of rosemary essential oil. This finding suggests that EOs can be used as a pre-emergent bioherbicide in the control of weeds [69]. The application of basil essential oil to fruits infected with *Monilinia fructicola* and *Prunus persica* var. *nucipersica* (Rosaceae) resulted in the inhibition of fungal growth [70]. The application of the basil essential oil effectively prevented the development of rot disease and demonstrated no significant phytotoxicity or adverse effects on the fruits. This study demonstrates that basil essential oil is an effective control measure for *P. oleracea*, with no significant impact on the germination of *G. max* and *Z. mays*, among other outcomes. Salvia essential oil was found to be highly phytotoxic to *Bromus secalinus* L. (Poaceae) and *Centaurea cyanus* L. (Asteraceae), with EC_50_ values of 0.02 g/L and 0.01 g/L, respectively [71]. The phytotoxicity of EOs is also inextricably contingent upon their chemical composition. The EO of *Artemisia absinthium* L. (Asteraceae) and its primary component, linalool, have been observed to exert an inhibitory effect on the root elongation of four plants, including *A. retroflexu*s [72].

In the future, additional toxicological experiments will be conducted on the primary chemical components of EOs to identify the chemicals that are toxic to thrips. The phytotoxicity of these major components to economic crops and weeds should also be determined. This will provide a basis for the development of botanical pesticides and behavioral regulators.

## 4. Materials and Methods

### 4.1. Essential Oils

The five Lamiaceae essential oils selected were melissa (*Melissa officinalis* L.), basil (*Ocimum gratissimum* L.), rosemary (*Rosmarinus officinalis* L.), negundo chastetree (*Vitex negundo* var. *cannabifolia* (Sieb. et Zucc.) Hand.-Mazz.), and salvia (*Salvia japonica* Thunb.). All the essential oils were obtained from Ji’an Zhongxiang Natural Plant Co., Ltd. (Ji’an city, Jiangxi Province, China). The extraction method used was steam distillation, and the purity of the essential oils was 98%.

### 4.2. Insect

In the experiment, *T. flavus* was collected from experimental fields with planted soybean in Changchun (43°48′8″ N, 125°24′32″ E), captured using sweeping nets, brought back to the laboratory, and then put in an incubator at a temperature of 25 ± 1 °C, with 70% ± 5% R.H. and a 16 h light to 8 h dark photoperiod, and the insects were fed soybean seedlings for two to three days [11,16]. The soybean seedlings were watered two to four times per week. Three-day-old thrips adults were used in the subsequent experiments.

### 4.3. Chemical Composition Analysis

The chemical composition of the Lamiaceae essential oils was analyzed using gas chromatography–mass spectrometry (GC–MS QP2010 plus, Shimadzu, Kyoto, Japan) equipped with a DB-5 capillary column (30 m × 0.25 mm i.d., 0.25 µm). The inlet was injected in the splitless mode, with a constant helium flow at a flow rate of 1 mL/min. The heating procedure followed the method described by Pei et al. [73]. The compound identification was carried out using GC–MS software applications, including the mass spectral libraries NIST 27 and NIST 147. The retention index was determined on the DB–5 capillary column using a homologous series of *n*-hydrocarbons [74].

### 4.4. Laboratory Bioassay

The laboratory toxicity was determined using the leaf-dipping method [73]. All five essential oils were diluted with acetone (Tianjin Xintong Fine Chemical Co., Ltd., Tianjin, China, 99.5% purity) to five concentrations of 0.2, 0.4, 0.6, 0.8, and 1.0 mg/mL. Fresh soybean leaves of the same size, without any damage, pests, or diseases, were carefully selected, washed with water, and allowed to dry naturally. The soybean leaves were immersed in the reagent solution for testing, air-dried at room temperature for 10 s, and then placed in a 50 mL plastic centrifuge tube with moisturizing filter paper. Each concentration treatment was replicated three times, with 30 *T. flavus* adults per replication. Additionally, 45% malathion EC, purchased from Hebei Jindelun Biochemical Technology Co., Ltd., Tangshan, China (Pesticide Registration Certificate No.: PD20084211), was used as an insecticide control. The survival status of *T. flavus* was examined after 24 h. The total number of thrips and the number of dead thrips were observed using a stereomicroscope (SZ61, Olympus Corporation, Tokyo, Japan) and recorded.

The mortality and adjusted mortality rates were calculated using the following equations:

(1) $$M_{1}=\frac{N_{1}}{N_{2}}\times 100$$

(2) $$M_{2}=\frac{MR_{2}-MR_{1}}{1-MR_{1}}\times 100$$

Here, *M*_1_ is the mortality rate; *N*_1_ is the number of dead thrips; *N*_2_ is the total number of thrips in the treatment; *M*_2_ is the adjusted mortality rate; *MR*_1_ is the mortality rate of the control; and *MR*_2_ is the mortality rate in the treatment.

### 4.5. Olfactory Behavior Test

The test procedure was modified based on the method described by Zhang et al. [75]. A Y-tube olfactometer was used to assess the olfactory behavioral responses of *T. flavus* to the plant essential oils. The selected essential oil was diluted to 1.0 mg/mL with acetone (Tianjin Xintong Fine Chemical Co., Ltd., 99.5% purity). Subsequently, 1 µL of the essential oil solution was added dropwise onto a piece of filter paper (length × width: 1 cm × 1 cm) placed in an odor-source bottle, with 1 µL of acetone used as the control. The Y-tube was connected to a vacuum pump blowing at a flow rate of 0.3 L/min into the Y-tube. To maintain consistent light conditions, the Y-tube was placed in a light box during the test, with an average light intensity of 7800–8000 lx. Each adult was observed for up to 5 min. When the test insect moved 1/2 of either arm of the Y-tube, it was considered to have made a choice. If no choice was made by the tested adults within 5 min, it was recorded as no choice. The test time ranged from 9:00 to 18:00. The room temperature ranged from 24 °C to 25 °C, and the relative humidity was between 30% and 40%. At least 30 adult female and 30 adult male thrips were tested for each essential oil. The gender of thrips can be determined by observing the morphology of the genitalia at the end of the abdomen under a stereomicroscope. Adult female thrips have ovipositors, while males do not [16]. The number of thrips in the selected treatment and control groups was recorded, and the lure and repellency rates were calculated.

The seduction and repellency rates were calculated using the following equations:

(3) $$S=\frac{C_{1}}{N}\times 100$$

(4) $$R=\frac{C_{2}}{N}\times 100$$

Here, *S* is the seduction rate; *C*_1_ is the number of thrips that selected the essential oil; *N* is the number of thrips that responded; *R* is the repellency rate; and *C*_2_ is the number of thrips that selected the control.

### 4.6. Pot Experiments

The pot experiments were performed using the method described by Pei et al. [73]. When the soybean plants developed their second compound leaf, the pots with the same growth were selected, and one soybean seedling without pests or diseases was kept in each pot. According to the results of the insecticidal activity determination, five doses (180.00 g a.i.·hm^−2^, 360.00 g a.i.·hm^−2^, 540.00 g a.i.·hm^−2^, 720.00 g a.i.·hm^−2^, and 900.00 g a.i.·hm^−2^) were set for each essential oil. The control group was sprayed with an acetone solution without the EOs. Each pot was sprayed evenly (5 mL) using a spray bottle. After the sprayed liquid was completely dried, the pot was covered with a 120-mesh gauze. Then, thirty adult thrips were introduced into each pot. The treatment pots were placed 1 m apart in a randomized block arrangement and raised under indoor conditions. At one day, three days, and seven days after spraying, the number of live thrips was observed and recorded.

The control efficacy was used to measure the ability of the insecticides or potential substitutes to control pests. The control efficacy was calculated using the following equation [21]:

(5) $$CE=\left(1-\frac{T_{1}\times C_{2}}{T_{2}\times C_{1}}\right)\times 100$$

Here, *CE* is the control efficacy, *T*_1_ is the live number of thrips in the treatment group after the treatment with the EOs, *T*_2_ is the live number of thrips in the treatment group before the treatment with the EOs, *C*_1_ is the live number of thrips in the control group after the treatment with the EOs, and *C*_2_ is the live number of thrips in the control group before the treatment with the EOs.

### 4.7. Phytotoxic Activity of Essential Oils

A phytotoxicity test was carried out using the method described in [76]. The five EOs were mixed with Tween 80 (Sinopharm Chemical Reagent Co., Ltd., Shanghai, China) in a 1:1 (*v*/*v*) ratio. The mixture was diluted with distilled water to five concentrations of 0.2, 0.4, 0.6, 0.8, and 1.0 mg/mL, and a distilled water solution of Tween 80 was used as a control. A total of two crops and two weeds were tested for phytotoxicity: *Glycine max* (L.) Merr., *Zea mays* L., *Portulaca oleracea* L., and *Echinochloa oryzoides* (Ard.) Fritsch, respectively. The seeds of each of the four crops were sterilized with 1% sodium hypochlorite (Guangdong Wenglong Biotechnology Co., Ltd., Guangzhou, China). Subsequently, they were rinsed 3 times using distilled water and filtered, and the sterilized seeds were placed in a glass Petri dish. Then, 30 seeds were placed in each Petri dish, and the essential oil solutions were added to the Petri dishes (25 mL solution was added to soybean and corn, and 2 mL solution was added to amaranth and barnyardgrass). Three replicates were set up for each concentration. The seeds were moisturized by laying a piece of filter paper on the top and bottom of each layer, and the filter paper was sprayed with the corresponding concentration of essential oil solution daily to keep the filter paper moist. The seed germination standard refers to the length of the embryonic root breaking through the seed coat, which should be 1 mm. All the treatments were incubated in a fully darkened artificial climatic chamber at a temperature of 25 ± 1 °C with 70 ± 5% relative humidity. The germination of the seeds in the Petri dishes was observed daily, and the number of germinated seeds per day was recorded for 7 d. The seed shoot length (SL) was measured using vernier calipers on day 7. The germination rate, germination potential, and germination index were calculated.

The germination rate, germination potential, and germination index were calculated using the following equation:

(6) $$GR=\frac{G_{1}}{G_{0}}\times 100$$

(7) $$GP=\frac{G_{2}}{G_{0}}\times 100$$

(8) $$GI=\sum \frac{Gt}{Dt}$$

Here, *GR* is the germination rate, *G*_1_ is the number of germinated seeds, *G*_0_ is the total number of seeds, *GP* is the germination potential, *G*_2_ is the number of germinated seeds on day 3, *GI* is the germination index, *Gt* is the number of germinated seeds on day *t*, and *Dt* is days to germination.

### 4.8. Data Analysis

The DPS 9.50 software (Hangzhou Ruifeng Information Technology Co., Ltd., Hangzhou, China, http://www.dpsw.cn, accessed on 9 March 2024) was used to fit the toxicity regression equation in order to obtain the correlation coefficients, LC_50_ values, and 95% confidence intervals [77]. The IBM SPSS statistics software (version 23.0) was used to perform a one-way ANOVA of the potting control efficacy and phytotoxicity results. Tukey’s test was used to compare significant differences between the treatments. The chi-square test was used to compare significant differences between the treatment and control groups in the olfactory behavior test. The figures were made using GraphPad Prism 9.5 (GraphPad Software, Boston, MA, USA).

## 5. Conclusions

*T. flavus* female adults were attracted to the negundo chastetree essential oil, which showed strong toxicity; thus, it has the potential for use as a botanical insecticide against *T. flavus*. The basil essential oil was equally toxic to *T. flavus* and was inhibitory to *P. oleracea* and, thus, has the potential to be developed as a biogenic herbicide. These EOs are safe for soybeans and corn. The results show that the major volatile components of the five Lamiaceae EOs preliminary analyzed were terpenoids, including *β*-pinene, *d*-limonene, *γ*-terpinene, and *α*-pinene. Compounds such as *β*-pinene and (+)-limonene are valuable for further studies as insecticide precursor compounds.

## Figures and Tables

**Table 1:** Chemical composition of salvia essential oil.

Number	Retention Index	Relative Percentage (%)	Name of Constituent
1	934	1.2	*α*-pinene
2	973	0.4	*β*-pinene
3	1013	2.2	*p*-cymene
4	1021	7.3	1,8-cineole
5	1079	0.5	terpinolene
6	1085	14.8	linalool
7	1126	4.1	camphor
8	1171	1.1	terpinen-4-ol
9	1179	2.6	*α*-terpineol
10	1208	1.2	7-methoxy-3,7-dimethyloctanal
11	1246	19.3	linalyl acetate
12	1338	0.4	linalyl anthranilate
13	1346	0.5	geranyl acetate
14	1364	0.7	4-Hexen-1-ol,5-methyl-2-(1-methylethenyl)-,acetate;2-isopropenyl-5-methylhex-4-enyl acetate
15	1418	0.5	*β*-caryophyllene
16	1434	0.5	*α*-muurolene
17	1480	0.4	germacrene D
18	1504	0.6	*α*-bulnesene
19	1654	1.1	patchouli alcohol
20	1728	12.3	benzyl benzoate
21	1813	28.4	isopropyl myristate

**Table 2:** Chemical composition of rosemary essential oil.

Number	Retention Index	Relative Percentage (%)	Name of Constituent
1	923	0.5	tricyclene
2	934	26.2	*α-*pinene
3	947	10.4	camphene
4	973	1.6	*β*-pinene
5	983	1.4	myrcene
6	998	1.4	*α-*phellandrene
7	1010	1.2	*α-*terpinene
8	1013	4.5	*p*-cymene
9	1021	25.9	1,8-cineole
10	1051	0.7	*γ*-terpinene
11	1085	0.9	linalool
12	1126	6.4	camphor
13	1159	4.8	borneol
14	1171	1.1	terpinen-4-ol
15	1179	2.0	*α*-terpineol
16	1185	5.2	(−)-verbenol
17	1273	2.2	bornyl acetate
18	1420	2.4	*β*-caryophyllene
19	1454	1.2	*α*-caryophyllene

**Table 3:** Chemical composition of basil essential oil.

Number	Retention Index	Relative Percentage (%)	Name of Constituent
1	1334	50.2	5-allylguaiacol
2	1347	1.6	thymoquinone
3	1354	0.8	isosafrole
4	1374	1.2	methyl eugenol
5	1379	12.1	*α*-copaene
6	1390	2.9	*β*-elemene
7	1397	1.1	benzene, [[(2,6,6-trimethyl-1-cyclohexen-1-yl)methyl]sulfonyl]-
8	1412	1.1	*α*-guaiene
9	1420	21.9	bicyclo[5.2.0]nonane, 4-ethenyl-4,8,8-trimethyl-2-methylene-
10	1430	0.8	(−)-alloaromadendrene
11	1434	1.5	*α*-bergamotene
12	1454	5.0	*α*-caryophyllene

**Table 4:** Chemical composition of negundo chastetree essential oil.

Number	Retention Index	Relative Percentage (%)	Name of Constituent
1	934	0.9	*α*-pinene
2	968	0.5	*β*-phellandrene
3	973	32.9	*β*-pinene
4	1013	9.6	benzene, 1-methyl-2-(1-methylethyl)-
5	1023	27.8	*d*-limonene
6	1039	3.4	ocimene
7	1051	16.9	*γ*-terpinene
8	1079	3.0	terpinolene
9	1332	3.2	eugenol
10	1381	1.8	*β*-caryophyllene

**Table 5:** Chemical composition of melissa essential oil.

Number	Retention Index	Relative Percentage (%)	Name of Constituent
1	1322	0.9	ionone
2	1365	1.2	*β*-vatirenene
3	1404	25.5	*β*-cadinene
4	1464	3.4	1,4-methano-1H-indene
5	1488	4.0	*α*-guaiene
6	1528	4.2	spathulenol
7	1605	2.2	2-naphthalenemethanol, 1,2,3,4,4a,8a-hexahydro-α,α,4a,8-tetramethyl-, (2R,4aS,8aR)-
8	1611	4.5	5(1H)-azulenone
9	1660	5.9	(+)-viridiflorol
10	1680	29.6	isolongifolen-5-one
11	1732	12.1	aristolone
12	1799	2.2	cyclodeca[b]furan-2(3H)-one
13	2191	4.2	1-phenanthrenecarboxylic acid

**Table 6:** LC_50_ values of essential oils from five essential oils against *Thrips flavus* in laboratory bioassays.

Essential Oils	Regression Equation	Correlation Coefficient	LC_50_ (mg/mL)	95% Confidence Interval	*χ* ^2^	*df*
Salvia essential oil	*y* = 6.3887 + 3.6539*x*	0.92	0.42	0.34~0.49	8.30	3
Rosemary essential oil	*y* = 6.5248 + 3.5398*x*	0.98	0.37	0.29~0.44	1.56	3
Basil essential oil	*y* = 7.3010 + 5.0998*x*	0.81	0.35	0.13~0.49	16.42	3
Negundo chastetree essential oil	*y* = 6.3913 + 3.0047*x*	0.79	0.34	0.26~0.42	5.09	3
Melissa essential oil	*y* = 6.8547 + 2.4828*x*	0.81	0.18	0.07~0.26	2.15	3
45% Malathion EC	*y =* 9.7959 + 2.5307*x*	0.94	0.0127	0.0068~0.0168	1.97	3

**Table 7:** Phytotoxic activity of salvia essential oil to four plants.

Plants	Concentration (mg/mL)	Germination Rate (%)	Germination Potential (%)	Germination Index	Shoot Length (mm)
*Glycine max*	control	100.00 ± 0 ^a^	100.00 ± 0 ^a^	51.42 ± 4.76 ^a^	62.18 ± 7.14 ^a^
	0.2	96.59 ± 0.14 ^b^	95.40 ± 2.20 ^c^	54.19 ± 2.78 ^a^	30.99 ± 2.55 ^c^
	0.4	96.63 ± 0.07 ^b^	96.63 ± 0.07 ^bc^	51.83 ± 3.06 ^a^	43.06 ± 3.64 ^bc^
	0.6	98.89 ± 1.92 ^a^	98.89 ± 1.92 ^ab^	52.75 ± 4.02 ^a^	44.17 ± 9.07 ^bc^
	0.8	96.67 ± 0 ^b^	96.67 ± 0 ^bc^	51.53 ± 2.47 ^a^	49.38 ± 7.28 ^ab^
	1.0	100.00 ± 0 ^a^	100.00 ± 0 ^a^	51.59 ± 5.89 ^a^	45.05 ± 2.96 ^bc^
*Zea mays*	control	95.48 ± 2.06 ^a^	88.77 ± 1.83 ^a^	40.30 ± 1.03 ^a^	14.77 ± 1.74 ^a^
	0.2	93.25 ± 8.78 ^a^	86.55 ± 8.76 ^a^	39.00 ± 3.13 ^a^	12.57 ± 5.67 ^a^
	0.4	91.03 ± 3.78 ^a^	82.22 ± 6.94 ^a^	37.33 ± 0.78 ^a^	14.42 ± 3.74 ^a^
	0.6	97.78 ± 3.85 ^a^	84.44 ± 9.62 ^a^	38.09 ± 1.54 ^a^	15.69 ± 5.57 ^a^
	0.8	90.00 ± 6.67 ^a^	86.67 ± 8.82 ^a^	35.51 ± 2.33 ^a^	10.86 ± 2.37 ^a^
	1.0	83.33 ± 10 ^a^	78.89 ± 7.70 ^a^	35.29 ± 4.20 ^a^	16.92 ± 4.84 ^a^
*Portulaca oleracea*	control	95.56 ± 1.92 ^a^	94.44 ± 3.85 ^a^	72.07 ± 1.69 ^a^	36.68 ± 2.66 ^ab^
	0.2	94.44 ± 1.92 ^a^	94.44 ± 1.92 ^a^	73.13 ± 1.85 ^a^	42.19 ± 4.17 ^a^
	0.4	96.67 ± 0 ^a^	96.67 ± 0 ^a^	74.19 ± 1.73 ^a^	35.57 ± 2.26 ^ab^
	0.6	95.56 ± 1.92 ^a^	91.11 ± 6.94 ^a^	69.55 ± 7.64 ^a^	35.28 ± 1.52 ^ab^
	0.8	96.67 ± 5.77 ^a^	86.67 ± 3.33 ^a^	67.86 ± 2.55 ^a^	32.21 ± 2.48 ^b^
	1.0	97.78 ± 1.92 ^a^	91.11 ± 5.09 ^a^	71.56 ± 3.07 ^a^	29.96 ± 3.43 ^b^
*Echinochloa oryzoides*	control	56.67 ± 10.00 ^a^	27.78 ± 9.62 ^a^	17.43 ± 1.58 ^a^	20.76 ± 9.00 ^a^
	0.2	58.89 ± 10.18 ^a^	32.22 ± 7.70 ^a^	17.11 ± 2.03 ^a^	30.13 ± 11.07 ^a^
	0.4	50.00 ± 13.33 ^a^	18.89 ± 13.47 ^a^	12.69 ± 5.64 ^a^	19.13 ± 10.67 ^a^
	0.6	58.89 ± 6.94 ^a^	16.67 ± 6.67 ^a^	13.55 ± 3.87 ^a^	23.9 ± 16.05 ^a^
	0.8	47.78 ± 1.92 ^a^	20.00 ± 6.67 ^a^	11.95 ± 1.74 ^a^	25.36 ± 10.06 ^a^
	1.0	48.89 ± 9.62 ^a^	24.44 ± 6.94 ^a^	12.53 ± 2.28 ^a^	5.94 ± 2.82 ^a^

Note: For the same EO, different lowercase letters following the data in the same column indicate significant differences in the five doses (*p* < 0.05). The same below.

**Table 8:** Phytotoxic activity of negundo chastetree essential oil to four plants.

Plants	Concentration (mg/mL)	Germination Rate (%)	Germination Potential (%)	Germination Index	Shoot Length (mm)
*Glycine max*	control	100.00 ± 0 ^a^	100.00 ± 0 ^a^	51.42 ± 4.76 ^a^	62.18 ± 7.14 ^a^
	0.2	100.00 ± 0 ^a^	100.00 ± 0 ^a^	60.72 ± 7.15 ^a^	52.73 ± 7.00 ^a^
	0.4	100.00 ± 0 ^a^	100.00 ± 0 ^a^	54.12 ± 1.61 ^a^	50.61 ± 9.20 ^a^
	0.6	100.00 ± 0 ^a^	98.89 ± 1.92 ^a^	52.51 ± 2.36 ^a^	64.33 ± 3.43 ^a^
	0.8	97.78 ± 3.85 ^a^	97.78 ± 3.85 ^ab^	55.39 ± 7.88 ^a^	55.39 ± 6.69 ^a^
	1.0	98.89 ± 1.92 ^a^	92.22 ± 1.92 ^b^	52.09 ± 6.59 ^a^	54.99 ± 3.45 ^a^
*Zea mays*	control	95.48 ± 2.06 ^a^	88.77 ± 1.83 ^ab^	40.3 ± 1.03 ^a^	14.77 ± 1.74 ^a^
	0.2	97.53 ± 4.28 ^a^	95.15 ± 5.69 ^a^	39.38 ± 3.94 ^a^	17.42 ± 4.67 ^a^
	0.4	96.67 ± 5.77 ^a^	89.43 ± 6.11 ^ab^	37.77 ± 1.85 ^a^	14.92 ± 5.94 ^a^
	0.6	94.21 ± 1.92 ^a^	82.59 ± 3.21 ^ab^	36.83 ± 0.59 ^a^	16.09 ± 7.53 ^a^
	0.8	97.78 ± 3.85 ^a^	81.29 ± 4.53 ^ab^	36.96 ± 0.48 ^a^	15.53 ± 4.85 ^a^
	1.0	96.67 ± 3.33 ^a^	76.67 ± 8.82 ^b^	37.79 ± 2.31 ^a^	9.90 ± 1.32 ^a^
*Portulaca oleracea*	control	95.56 ± 1.92 ^a^	94.44 ± 3.85 ^a^	72.07 ± 1.69 ^a^	36.68 ± 2.66 ^abc^
	0.2	98.89 ± 1.92 ^a^	95.56 ± 5.09 ^a^	61.59 ± 7.53 ^a^	38.72 ± 2.27 ^a^
	0.4	95.56 ± 3.85 ^a^	87.78 ± 8.39 ^a^	61.72 ± 5.50 ^a^	38.10 ± 2.78 ^ab^
	0.6	93.33 ± 3.33 ^a^	90.00 ± 3.33 ^a^	61.85 ± 5.54 ^a^	27.59 ± 3.99 ^c^
	0.8	93.33 ± 3.33 ^a^	88.89 ± 1.92 ^a^	68.99 ± 0.05 ^a^	28.95 ± 5.70 ^bc^
	1.0	96.67 ± 5.77 ^a^	96.67 ± 5.77 ^a^	74.19 ± 3.92 ^a^	40.29 ± 1.97 ^a^
*Echinochloa oryzoides*	control	56.67 ± 10.00 ^a^	27.78 ± 9.62 ^a^	17.43 ± 1.58 ^a^	20.76 ± 9.00 ^a^
	0.2	41.11 ± 3.85 ^a^	23.33 ± 8.82 ^a^	12.05 ± 2.94 ^a^	25.45 ± 16.58 ^a^
	0.4	45.56 ± 11.71 ^a^	17.78 ± 11.71 ^a^	13.55 ± 2.60 ^a^	13.98 ± 2.09 ^a^
	0.6	55.56 ± 10.18 ^a^	37.78 ± 11.71 ^a^	18.8 ± 4.45 ^a^	18.27 ± 6.73 ^a^
	0.8	46.67 ± 11.55 ^a^	24.44 ± 5.09 ^a^	13.37 ± 1.28 ^a^	20.27 ± 13.00 ^a^
	1.0	57.78 ± 1.92 ^a^	34.44 ± 10.18 ^a^	16.90 ± 0.83 ^a^	19.68 ± 5.66 ^a^

Note: For the same EO, different lowercase letters following the data in the same column indicate significant differences in the five doses (*p* < 0.05). The same below.

**Table 9:** Phytotoxic activity of basil essential oil to four plants.

Plants	Concentration (mg/mL)	Germination Rate (%)	Germination Potential (%)	Germination Index	Shoot Length (mm)
*Glycine max*	control	100.00 ± 0 ^a^	100.00 ± 0 ^a^	51.42 ± 4.76 ^a^	62.18 ± 7.14 ^a^
	0.2	100.00 ± 0 ^a^	94.44 ± 5.09 ^b^	51.73 ± 2.28 ^a^	41.32 ± 6.20 ^b^
	0.4	100.00 ± 0 ^a^	100.00 ± 0 ^a^	50.29 ± 1.73 ^a^	49.42 ± 6.23 ^ab^
	0.6	100.00 ± 0 ^a^	100.00 ± 0 ^a^	50.92 ± 1.18 ^a^	48.88 ± 4.81 ^ab^
	0.8	100.00 ± 0 ^a^	100.00 ± 0 ^a^	48.29 ± 2.18 ^a^	41.57 ± 7.68 ^b^
	1.0	100.00 ± 0 ^a^	100.00 ± 0 ^a^	48.69 ± 4.86 ^a^	38.40 ± 11.18 ^b^
*Zea mays*	control	95.48 ± 2.06 ^a^	88.77 ± 1.83 ^a^	40.30 ± 1.03 ^a^	14.77 ± 1.74 ^a^
	0.2	93.33 ± 3.33 ^a^	84.44 ± 10.72 ^a^	39.13 ± 2.32 ^ab^	15.09 ± 6.51 ^a^
	0.4	95.56 ± 1.92 ^a^	76.67 ± 3.33 ^a^	37.76 ± 2.62 ^abc^	11.37 ± 1.94 ^a^
	0.6	91.87 ± 4.16 ^a^	83.22 ± 6.50 ^a^	37.36 ± 1.02 ^abc^	9.85 ± 1.69 ^a^
	0.8	93.30 ± 3.28 ^a^	79.37 ± 2.74 ^a^	34.36 ± 1.45 ^c^	10.98 ± 2.24 ^a^
	1.0	91.11 ± 1.92 ^a^	86.67 ± 5.77 ^a^	35.43 ± 0.95 ^bc^	13.73 ± 1.72 ^a^
*Portulaca oleracea*	control	95.56 ± 1.92 ^a^	94.44 ± 3.85 ^a^	72.07 ± 1.69 ^a^	36.68 ± 2.66 ^b^
	0.2	98.89 ± 1.92 ^a^	98.89 ± 1.92 ^a^	76.59 ± 1.31 ^a^	45.62 ± 0.19 ^a^
	0.4	96.67 ± 0 ^a^	96.67 ± 0 ^a^	69.19 ± 0 ^a^	19.70 ± 2.49 ^c^
	0.6	48.89 ± 13.47 ^b^	42.22 ± 8.39 ^b^	26.36 ± 6.82 ^b^	1.76 ± 0.52 ^d^
	0.8	38.89 ± 13.47 ^b^	13.33 ± 14.53 ^bc^	11.44 ± 6.41 ^c^	1.40 ± 0.24 ^d^
	1.0	11.11 ± 5.09 ^c^	6.67 ± 8.82 ^c^	4.00 ± 3.60 ^c^	1.33 ± 0.11 ^d^
*Echinochloa oryzoides*	control	56.67 ± 10.00 ^a^	27.78 ± 9.62 ^a^	17.43 ± 1.58 ^ab^	20.76 ± 9.00 ^a^
	0.2	64.44 ± 15.75 ^a^	36.67 ± 8.82 ^a^	17.89 ± 5.50 ^a^	17.84 ± 15.63 ^a^
	0.4	46.67 ± 6.67 ^ab^	18.89 ± 5.09 ^ab^	11.08 ± 2.38 ^abc^	16.94 ± 4.56 ^a^
	0.6	45.56 ± 6.94 ^ab^	16.67 ± 11.55 ^ab^	9.89 ± 2.57 ^bc^	13.75 ± 7.69 ^a^
	0.8	42.22 ± 13.47 ^ab^	14.44 ± 1.92 ^ab^	8.30 ± 0.97 ^c^	5.55 ± 2.73 ^a^
	1.0	31.11 ± 6.94 ^b^	6.67 ± 3.33 ^b^	5.46 ± 1.08 ^c^	4.21 ± 4.81 ^a^

Note: For the same EO, different lowercase letters following the data in the same column indicate significant differences in the five doses (*p* < 0.05). The same below.

**Table 10:** Phytotoxic activity of rosemary essential oil to four plants.

Plants	Concentration (mg/mL)	Germination Rate (%)	Germination Potential (%)	Germination Index	Shoot Length (mm)
*Glycine max*	control	100.00 ± 0 ^a^	100 00 ± 0 ^a^	51.42 ± 4.76 ^a^	62.18 ± 7.14 ^a^
	0.2	100.00 ± 0 ^a^	100.00 ± 0 ^a^	55.72 ± 3.82 ^a^	42.71 ± 13.57 ^ab^
	0.4	100.00 ± 0 ^a^	97.62 ± 4.12 ^a^	53.56 ± 2.05 ^a^	58.60 ± 5.65 ^ab^
	0.6	100.00 ± 0 ^a^	97.74 ± 1.96 ^a^	51.83 ± 0.63 ^a^	38.24 ± 5.40 ^b^
	0.8	100.00 ± 0 ^a^	100.00 ± 0 ^a^	50.59 ± 2.88 ^a^	62.34 ± 5.27 ^a^
	1.0	100.00 ± 0 ^a^	96.50 ± 0.19 ^a^	47.33 ± 1.56 ^a^	39.69 ± 3.56 ^b^
*Zea mays*	control	95.48 ± 2.06 ^a^	88.77 ± 1.83 ^a^	40.30 ± 1.03 ^a^	14.77 ± 1.74 ^a^
	0.2	97.78 ± 3.85 ^a^	82.18 ± 13.41 ^a^	39.64 ± 2.72 ^a^	12.18 ± 4.20 ^a^
	0.4	98.89 ± 1.92 ^a^	94.33 ± 4.05 ^a^	38.53 ± 2.76 ^a^	9.42 ± 3.82 ^a^
	0.6	89.41 ± 10.72 ^a^	81.28 ± 16.22 ^a^	35.75 ± 5.33 ^a^	15.31 ± 3.97 ^a^
	0.8	94.25 ± 1.99 ^a^	83.91 ± 7.18 ^a^	36.52 ± 3.01 ^a^	13.96 ± 4.25 ^a^
	1.0	90.96 ± 7.83 ^a^	88.50 ± 7.98 ^a^	36.43 ± 1.11 ^a^	12.97 ± 2.82 ^a^
*Portulaca oleracea*	control	95.56 ± 1.92 ^a^	94.44 ± 3.85 ^a^	72.07 ± 1.69 ^a^	36.68 ± 2.66 ^bc^
	0.2	96.67 ± 3.33 ^a^	96.67 ± 3.33 ^a^	71.69 ± 1.78 ^a^	38.49 ± 1.43 ^b^
	0.4	96.67 ± 0 ^a^	96.67 ± 0 ^a^	71.03 ± 2.47 ^a^	43.39 ± 0.85 ^a^
	0.6	95.56 ± 1.92 ^a^	94.44 ± 1.92 ^a^	73.05 ± 1.96 ^a^	45.03 ± 0.39 ^a^
	0.8	94.44 ± 3.85 ^a^	94.44 ± 3.85 ^a^	71.80 ± 1.63 ^a^	34.87 ± 0.52 ^bc^
	1.0	96.67 ± 5.77 ^a^	86.67 ± 5.77 ^a^	69.69 ± 4.49 ^a^	33.25 ± 2.31 ^c^
*Echinochloa oryzoides*	control	56.67 ± 10.00 ^a^	27.78 ± 9.62 ^a^	17.43 ± 1.58 ^ab^	20.76 ± 9.00 ^a^
	0.2	52.22 ± 6.94 ^a^	28.89 ± 5.09 ^a^	14.67 ± 0.92 ^ab^	33.85 ± 5.91 ^a^
	0.4	61.11 ± 5.09 ^a^	34.44 ± 1.92 ^a^	18.14 ± 1.21 ^a^	36.4 ± 1.82 ^a^
	0.6	52.22 ± 6.94 ^a^	25.56 ± 5.09 ^a^	13.88 ± 2.07 ^ab^	17.46 ± 4.89 ^a^
	0.8	53.33 ± 14.53 ^a^	26.67 ± 8.82 ^a^	14.67 ± 3.99 ^ab^	19.38 ± 6.79 ^a^
	1.0	45.56 ± 5.09 ^a^	22.22 ± 1.92 ^a^	12.45 ± 0.72 ^c^	21.80 ± 11.80 ^a^

Note: For the same EO, different lowercase letters following the data in the same column indicate significant differences in the five doses (*p* < 0.05). The same below.

**Table 11:** Phytotoxic activity of melissa essential oil to four plants.

Plants	Concentration (mg/mL)	Germination Rate (%)	Germination Potential (%)	Germination Index	Shoot Length (mm)
*Glycine max*	control	100.00 ± 0 ^a^	100.00 ± 0 ^a^	51.42 ± 4.76 ^ab^	62.18 ± 7.14 ^a^
	0.2	100.00 ± 0 ^a^	92.22 ± 7.70 ^a^	53.01 ± 1.00 ^a^	47.77 ± 9.18 ^a^
	0.4	100.00 ± 0 ^a^	90.79 ± 7.99 ^a^	46.53 ± 1.73 ^ab^	49.39 ± 1.24 ^a^
	0.6	100.00 ± 0 ^a^	89.69 ± 6.95 ^a^	43.06 ± 2.47 ^b^	43.66 ± 10.08 ^a^
	0.8	98.89 ± 1.92 ^a^	95.56 ± 1.92 ^a^	47.75 ± 4.99 ^ab^	50.71 ± 17.05 ^a^
	1.0	95.56 ± 3.85 ^a^	90.00 ± 3.33 ^a^	44.61 ± 2.46 ^ab^	36.77 ± 3.41 ^a^
*Zea mays*	control	95.48 ± 2.06 ^abc^	88.77 ± 1.83 ^ab^	40.30 ± 1.03 ^a^	14.77 ± 1.74 ^a^
	0.2	100.00 ± 0 ^a^	94.41 ± 6.92 ^a^	40.95 ± 1.59 ^a^	9.58 ± 2.42 ^b^
	0.4	89.57 ± 3.81 ^bc^	87.35 ± 2.06 ^ab^	37.19 ± 3.04 ^a^	17.02 ± 2.36 ^a^
	0.6	83.89 ± 10.37 ^c^	73.27 ± 0.83 ^b^	31.98 ± 2.73 ^a^	12.84 ± 1.10 ^ab^
	0.8	98.89 ± 1.92 ^ab^	88.49 ± 7.43 ^ab^	39.33 ± 6.31 ^a^	13.19 ± 0.69 ^ab^
	1.0	94.25 ± 7.18 ^abc^	80.65 ± 4.16 ^b^	36.27 ± 2.49 ^a^	13.84 ± 0.40 ^ab^
*Portulaca oleracea*	control	95.56 ± 1.92 ^a^	94.44 ± 3.85 ^a^	72.07 ± 1.69 ^a^	36.68 ± 2.66 ^a^
	0.2	97.78 ± 3.85 ^a^	94.44 ± 9.62 ^a^	71.22 ± 11.37 ^a^	33.49 ± 1.31 ^a^
	0.4	98.89 ± 1.92 ^a^	96.67 ± 5.77 ^a^	70.20 ± 8.33 ^a^	23.37 ± 1.02 ^b^
	0.6	92.22 ± 1.92 ^a^	86.67 ± 5.77 ^a^	64.68 ± 4.49 ^a^	21.40 ± 1.20 ^b^
	0.8	95.56 ± 7.70 ^a^	94.44 ± 9.62 ^a^	66.05 ± 4.31 ^a^	15.44 ± 1.28 ^c^
	1.0	97.78 ± 3.85 ^a^	94.44 ± 9.62 ^a^	70.26 ± 6.76 ^a^	11.69 ± 0.92 ^c^
*Echinochloa oryzoides*	control	56.67 ± 10.00 ^a^	27.78 ± 9.62 ^a^	17.43 ± 1.58 ^a^	20.76 ± 9.00 ^a^
	0.2	43.33 ± 17.64 ^a^	27.78 ± 5.09 ^a^	13.90 ± 4.12 ^a^	16.66 ± 6.55 ^a^
	0.4	57.78 ± 10.18 ^a^	22.22 ± 3.85 ^a^	15.15 ± 0.91 ^a^	12.90 ± 5.04 ^a^
	0.6	57.78 ± 5.09 ^a^	27.78 ± 1.92 ^a^	17.31 ± 0.14 ^a^	12.06 ± 2.89 ^a^
	0.8	47.78 ± 8.39 ^a^	17.78 ± 15.75 ^a^	12.27 ± 0.78 ^a^	6.29 ± 3.13 ^a^
	1.0	45.56 ± 5.09 ^a^	24.44 ± 8.39 ^a^	13.95 ± 0.68 ^a^	7.73 ± 0.96 ^a^

Note: For the same EO, different lowercase letters following the data in the same column indicate significant differences in the five doses (*p* < 0.05). The same below.

## Data Availability

Data are available within the article.
